# Oversizing Consideration of Proximal Stent Graft in Hemodynamically Stable and Unstable Patients Undergoing Emergent Endovascular Aortic Repair

**DOI:** 10.3390/jcm12237500

**Published:** 2023-12-04

**Authors:** Yuhan Qi, Chengxin Weng, Ding Yuan, Tiehao Wang, Yukui Ma, Yi Yang, Jichun Zhao, Bin Huang

**Affiliations:** 1 Division of Vascular Surgery, Department of General Surgery, West China Hospital, Sichuan University, Chengdu 610041, China; qiyuhan7777@163.com (Y.Q.); chengxin_weng@wchscu.cn (C.W.); yuanding@wchscu.cn (D.Y.); tiehao.wang@wchscu.cn (T.W.); mayukui@scu.edu.cn (Y.M.); yangy@wchscu.cn (Y.Y.); 2 West China School of Medicine, Sichuan University, Chengdu 610041, China

**Keywords:** aortic aneurysm, endovascular procedures, oversizing ratio, type IA endoleak

## Abstract

Consideration for oversizing the proximal stent graft is suggested in endovascular aortic repair. However, a special recommendation for the proximal oversizing ratio (OSR) in patients with ruptured abdominal aortic aneurysm (rAAA) is ambiguous. This study aims to evaluate the effect of different degrees of the proximal oversizing ratio (OSR) on risk of type IA endoleak (TIAEL) in hemodynamically stable and unstable patients with rAAA undergoing emergency endovascular aortic repair (EVAR). Our study included 134 rAAA patients undergoing emergent EVAR, and we did not observe a significant association between hemodynamic instability and risk of T1AEL (HR 3.89, 95%CI 0.40–37.75, *p* = 0.24). All three T1AELs in the hemodynamically unstable subgroup were observed in patients with OSR ≤ 30%, but no significant difference was found regarding T1AEL between patients with OSR > 30% and OSR ≤ 30% (0.00% vs. 11.11%, *p* = 0.19). As for hemodynamically stable patients, OSR > 20% was associated with a significantly decreased risk of T1AEL (HR 0.03, 95%CI 0.01–0.53, *p* = 0.016). In conclusion, a proximal OSR > 20% is associated with a decreased risk of T1AEL in hemodynamically stable patients, while an OSR > 30% did not add an additional advantage of lowering the risk of T1AEL in hemodynamically unstable patients.

## 1. Introduction

Since the introduction of endovascular aneurysm repair (EVAR) in 1991 [1], ruptured abdominal aortic aneurysm (rAAA) has increasingly been treated with EVAR owing to its potential short-term benefits on survival and morbidity [2]. Data from individual-patient meta-analysis of three randomized controlled trials showed comparable survival but earlier discharge after EVAR in rAAA patients. Thus EVAR is now widely accepted in appropriately selected rAAA patients when adequate personnel and resources are present [3,4]. However, a relatively higher incidence of aneurysm-related complications and reinterventions remains the main drawback of endovascular repair [5]. Notably, the most disturbing complication is considered to be type IA endoleak (T1AEL), which can cause considerable blood flow leakage into the aneurysmal sac and remains the most important reason for late aneurysm rupture after EVAR [6,7].

To ensure the long-term stability of the stent graft, sufficient fixation and sealing of the proximal anchoring segments are essential. According to current guidelines and most instructions for the use of elective EVAR, stent grafts are often oversized by 10% to 25% to provide adequate radial support force for proximal neck anchoring [8,9]. However, there is ambiguity regarding the specific recommendation for the proximal oversizing ratio (OSR) in rAAA patients undergoing EVAR. 

To avoid intraoperative or late type IA endoleak (T1AEL), the recent ESVS guideline suggests that a 30% oversizing of the stent graft in the proximal landing zone is preferable for patients undergoing emergency EVAR [8]. However, this recommendation was only inferred from a case report and a clinical observation of aortic diameter changes in trauma patients [10,11]. On the other hand, in vitro research indicated that excessive oversizing should also be avoided in the proximal anchoring, because oversizing >30% can lead to graft folding and poor barb penetration [12]. Clinical evidence also suggests endograft oversizing of >30% can significantly raise the risk of device migration in the midterm after EVAR [13,14]. Given the current rare and conflicting data, the investigation of the appropriate degree of proximal stent graft oversizing is necessary in emergency EVAR. 

Thus, the purpose of our study was to investigate the influence of different degrees of proximal OSR on the risk of T1AEL in hemodynamically stable and unstable rAAA patients undergoing emergency EVAR.

## 2. Materials and Methods

### 2.1. Study Design and Approvals

A retrospective cohort study was conducted based on a database of patients who underwent EVAR at a tertiary academic hospital with a high volume of aortic surgeries. This study was reported in accordance with the Strengthening the Reporting of Observational Studies in Epidemiology (STROBE) Statement of cohort studies [15]. The STROBE checklist is shown in Table S1. Ethical approval for this study was obtained from the Ethics Committee of West China Hospital of Sichuan University (approval number: 2023-1705).

### 2.2. Participants

All consecutive patients undergoing emergency EVAR for impending rupture or ruptured AAA between July 2010 and December 2020 were included. Ruptured AAA was defined as the presence of retroperitoneal and/or intraperitoneal hematoma adjacent to the aneurysm sac in a preoperative computed tomography angiogram (CTA). Impending rupture was defined as those who had an acute onset or exacerbation of abdominal pain, with at least one of the following CTA findings: draped aorta sign, well-defined peripheral crescent of increased attenuation within the thrombus of a large AAA, focal discontinuity in circumferential wall calcifications, or a collection of fluid around the aneurysm sac [16]. Ruptured abdominal aortic aneurysm mostly presents as retroperitoneal hematoma and abdominal aortic aneurysm. Blood around the abdominal aorta may extend into the perirenal space and pararenal space. A localized gap of discontinuity of the aortic wall or continuous calcification of the surrounding wall may point to the site of rupture [16]. All the diagnoses of rAAA were confirmed by agreement between two vascular surgeons based on CTA findings. Patients were excluded if they met the following criteria: (1) juxtarenal aortic aneurysm repaired by hybrid or chimney techniques; (2) pseudoaneurysms from trauma or infection; (3) reintervention for endoleaks of prior endovascular procedures; (4) proximal oversizing was not available due to the absence of preoperative CTA.

### 2.3. Exposures

Proximal oversizing ratio (OSR) of the stent graft was defined as the ratio of the diameter difference between the aneurysm neck and proximal stent graft to the diameter of the aneurysm neck. The stent graft adopted in our study was from one manufacturer, Endurant II or IIs Stent Graft (Medtronic, Minneapolis, MN, USA). All measurements of preoperative proximal sizing were performed by two experienced vascular surgeons on preoperative CTA. 

All included patients were divided into two subgroups based on the hemodynamic stability assessed by mean arterial pressure (MAP) and heart rate. Hemodynamic instability was defined as MAP ≤ 90 mmHg and heart rate ≥ 100 beats/min. The cutting points of proximal OSR of stent grafts were set as 20% and 30% in hemodynamically stable and unstable patients, respectively.

During the treatment, patients received either local or general anesthesia and systemic heparinization (intravenous injection of unfractionated heparin at a dose of 0.5 mg/kg). A vascular access sheath was inserted through percutaneous puncture or a femoral artery incision. Abdominal aortography was performed to evaluate the neck and morphology of the aneurysm. A 0.035 ‘Lunderquist^®^ (COOK^®^, Inc., Bloomington, IN, USA) superhard guidewire was inserted into the abdominal aortic aneurysm main stent delivery device, and the proximal end of the stent was placed horizontally on the lower edge of the lower renal artery for release. Angiography was repeated above the level of the renal artery to confirm the distance between the main bifurcation and the common iliac bifurcation. The iliac stent transporter was placed along a 0.035 ‘Lunderquist^®^ (COOK^®^, Inc., Bloomington, IN, USA) superhard guidewire. The proximal flat stent body bifurcation was marked, and the distal position was confirmed to be appropriate before releasing the iliac stent. Angiography was reviewed to confirm the presence of endoleak, and if necessary, a Reliant AB46 (Medtronic^®^, Inc., Minneapolis, MN, USA) balloon was applied to the neck, iliac branch, or stent junction, and cuff stents were added at the discretion of the surgeon. After confirming angiography, the guidewire catheter was withdrawn, and the blood flow of both lower limbs was opened to check the distal arterial pulse, comparing with that before the operation. All patients received fluid infusion and antiplatelet therapy with aspirin 100 mg/d after the operation.

### 2.4. Outcomes of Interest and Follow-Up Protocols

Patients undergoing EVAR were regularly followed up in outpatient clinics with vascular contrast-enhanced ultrasonography at 1, 6, and 12 months, and annually thereafter. CTA was performed if ultrasound observed T1AEL or other adverse aneurysm-related complications. Telephone calls were used to check the status and follow-up details of patients if they failed to come.

The primary outcome of interest was the rate of T1AEL after EVAR, identified by either duplex ultrasound or CTA during follow-up. Secondary outcomes included 30-day mortality, aneurysm-related reintervention, other types of endoleaks, and overall survival.

### 2.5. Variables

Demographics, comorbidities, vital signs on admission, aortic neck characteristics, and aneurysm-related parameters were recorded to address the potential confounding effect of covariates. The Charlson comorbidity index (CCI) was used to evaluate the severity of comorbidities in included patients [17]. Aortic neck parameters involved the aneurysmal neck diameter, neck length of the aneurysm, neck angulation (α and β angle according to previous definitions, [18]), and neck calcification of the aneurysm. CTA examinations in this study were acquired using a Philips Brilliance 64-slice spiral CT scanner. Data were post-processed on a Siemens workstation (Leonardo, Siemens Medical Solutions, Erlangen, Germany), including CT vascular three-dimensional reconstruction. All diameters were measured in the minor axis of axial cuts or planes perpendicular to the centerline of reformatted slices from adventitia to adventitia. Aneurysmal neck diameter was measured at the level immediately below the lowest renal artery. Neck calcification was defined as the circumferential proportion of calcified vessel wall at the aneurysm neck, and was estimated from the cross-sectional images. The maximum diameter of the aneurysm and concomitant common iliac artery aneurysm (CIAA) were also recorded. CIAA was defined as any aneurysmal dilation of the common iliac arteries with a diameter larger than 24 mm.

### 2.6. Statistical Analyses

All statistical analyses were performed using R Studio Version 1.2.1335 (http://www.R-project.org, accessed on 11 October 2023) and Empower(R) (www.empowerstats.com, accessed on 11 October 2023, X&Y solutions, Inc., Boston, MA, USA). Continuous data were compared with Student *t* test or Mann–Whitney U test and categorical data were compared using Fisher’s exact test or χ^2^ test. The Kaplan–Meier method was used to estimate the cumulative rate of time-to-event outcomes after EVAR, and log-rank tests were used to compare the differences in the estimated incidence of outcomes between groups. 

To address the confounding effect of covariates, multivariate logistic regression analysis was performed to evaluate the association between OSR and 30-day mortality, and Cox proportional hazard regression analysis was performed to evaluate the effect of OSR on time-to-event outcomes. Covariate selection in the multivariate models was based on the change-in-estimate criteria and clinical relevance [19]. Missing data were handled with multiple imputation. Two subgroup analyses were performed in hemodynamically stable and unstable patients. A *p* value < 0.05 was considered statistically significant.

## 3. Results

### 3.1. Baseline Characteristics

**Figure 1:**
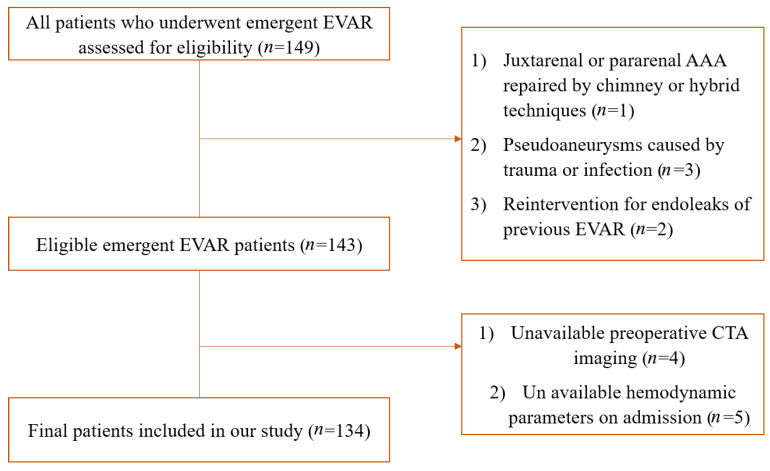
Flow diagram of selection of eligible patients.

There were 134 patients who underwent emergent EVAR for ruptured or impending rupture AAA. The detailed process of patient selection is displayed in Figure 1. Of these, 44 patients were considered hemodynamically unstable, with a mean MAP of 80.61 ± 9.30 mmHg and a mean heart rate of 103.82 ± 5.96 beats/min. The placement of the stent within indications for use was similar between hemodynamically stable and unstable patients (41.11% vs. 47.73%, *p* = 0.46). The mean age was comparable between hemodynamically stable and unstable patients (70.41 ± 9.89 vs. 72.55 ± 8.24, *p* = 0.22), while females were more common in hemodynamically unstable patients (36.36% vs. 18.89%, *p* = 0.027). The neck morphology was comparable between groups, except for hemodynamically unstable patients had larger α angles [27.66 (12.93–45.42) vs. 32.50 (10.77–66.73), *p* = 0.025]. The proximal OSR was significantly larger in hemodynamically unstable patients (25% (22–32%) vs. 22% (20–27%), *p* = 0.011). The baseline characteristics of included patients are summarized in Table 1.

### 3.2. Risk Factors of T1AEL in Emergent EVAR

During a median imaging follow-up of 13 (3.50–34.00) months, eight patients were found to have T1AEL. The results of univariate regression analysis suggested that larger neck diameter was associated with a significantly increased risk of T1AEL (HR 1.27, 95%CI 1.03–1.56, *p* = 0.028), while OSR > 20% (HR 0.06, 95%CI 0.01–0.31, *p* = 0.001) and longer neck length (HR 0.83, 95%CI 0.72–0.95, *p* = 0.009) was associated with a significantly decreased risk of T1AEL. After adjusted for gender, age, and neck angulation, only OSR > 20% (HR 0.06, 95%CI 0.01–0.72, *p* = 0.026) and longer neck length (HR 0.74, 95%CI 0.56–0.98, *p* = 0.033) remained significantly associated with the risk of T1AEL (Table 2).

### 3.3. Comparison of Outcomes in Hemodynamically Unstable and Stable Patients

The median survival (24.5 months vs. 33.0 months, *p* = 0.22) and imaging (10.0 months vs. 15.0 months, *p* = 0.24) follow-up time were similar between hemodynamically unstable and stable patients. We observed T1AEL in 3 (7.32%) hemodynamically unstable and 5 (5.56%) hemodynamically stable patients. As is shown in Table S2, no significant association was found between hemodynamic instability and the risk of T1AEL (HR 3.89, 95%CI 0.40–37.75, *p* = 0.24), as well as the risk of reintervention (HR 0.92, 95%CI 0.22–3.76, *p* = 0.90). However, hemodynamic instability was associated with a significantly increased risk of all-cause mortality (HR 2.12, 95%CI 1.06–4.27, *p* = 0.034). Higher MAP was associated with a decreased risk of 30-day mortality (β = 0.92, 95%CI 0.85–1.00, *p* = 0.038).

### 3.4. OSR > 30% versus OSR ≤ 30% in Hemodynamically Unstable Patients

**Figure 2:**
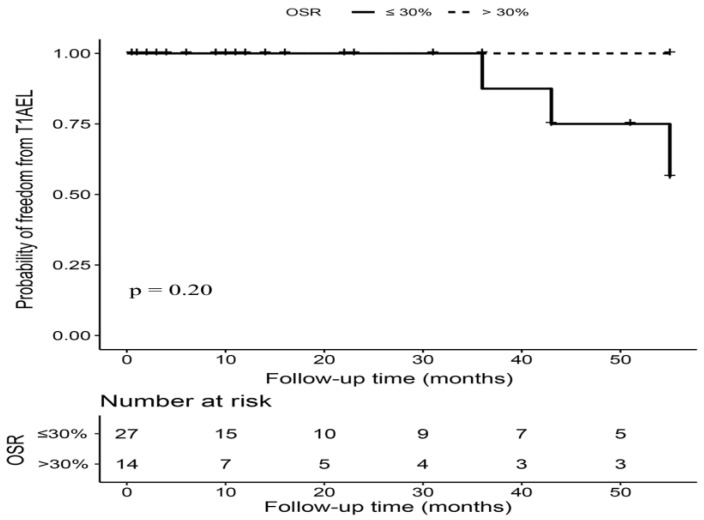
Kaplan–Meier curves of freedom from T1AEL in hemodynamically unstable patients with OSR > 30% versus OSR ≤ 30%. Footnote: T1AEL = type IA endoleak, OSR = oversizing ratio.

Among the 44 hemodynamically unstable patients, 14 (31.82%) patients had a proximal stent graft OSR over 30%. The mean proximal OSR was 26% ± 5%, range from 17% to 39%. The median imaging follow-up time was similar between patients with OSR ≤ 30% and OSR > 30% (10.50 (1.25–32.50) months vs. 10.00 (1.00–39.50), *p* = 0.87). All three T1AELs occurred in patients with OSR ≤ 30%, but we did not observe a significant difference in T1AEL between patients with OSR > 30% and OSR ≤ 30% (0.00% vs. 11.11%, *p* = 0.19). There was no early T1AEL; all the three T1AELs in hemodynamically unstable patients were observed after 36 months postintervention (Figure 2). Additionally, no significant difference was found regarding the rates of other types of endoleaks, reintervention, and survival outcomes (Table 3).

### 3.5. OSR > 20% versus OSR ≤ 20% in Hemodynamically Stable Patients

**Figure 3:**
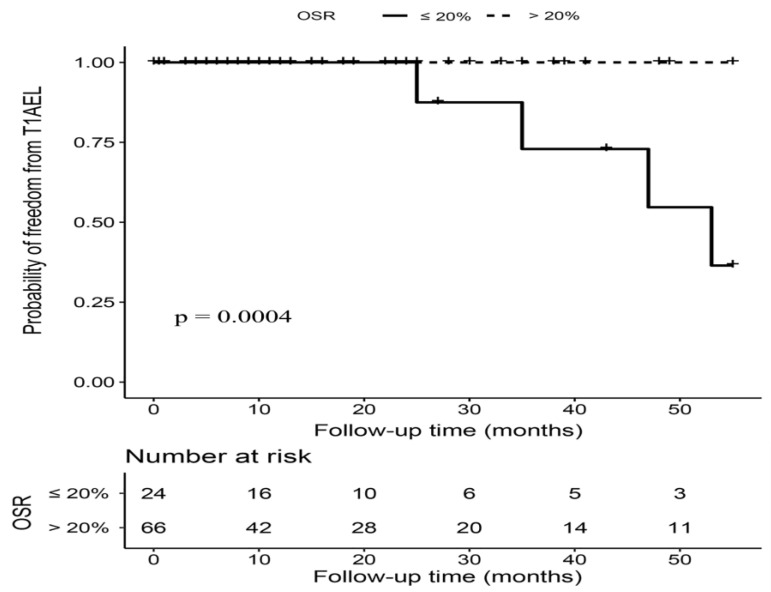
Kaplan–Meier curves of freedom from type IA endoleak (T1AEL) in hemodynamically stable patients with OSR > 20% versus OSR ≤ 20%. Footnote: T1AEL = type IA endoleak, OSR = oversizing ratio.

The proximal OSR ranged from 16% to 34%, with 66 (73.33%) patients having an OSR over 20%. Patients with OSR > 20% and OSR ≤ 20% had a similar length of imaging follow-up time (15.00 (5.25–34.50) months vs. 16.50 (5.00–29.00) months, *p* = 0.71). As is displayed in Figure 3, five T1AELs were observed after 24 months postintervention during the follow-up, and the incidence of T1AEL was significantly higher in patients with OSR ≤ 20% (16.67% vs. 1.52%, *p* = 0.006). After adjusted for age, gender, and neck angulation, OSR > 20% was associated with a significantly decreased risk of T1AEL in hemodynamically stable patients (HR 0.03, 95%CI 0.01–0.53, *p* = 0.016). The rates of other types of endoleaks, reintervention, and survival outcomes were comparable between two OSR groups (Table 3).

## 4. Discussion

This cohort study reviewed all patients who underwent emergent EVAR in a high-volume center in the past decade. The results suggested that a proximal OSR over 20% was associated with a significantly decreased risk of T1AEL after emergent EVAR. With a mean OSR of 26%, hemodynamic instability was not associated with a significantly increased risk of T1AEL. As for hemodynamically unstable patients, a proximal OSR over 30% did not seem to exert additional benefit in lowering the risk of T1AEL. Most T1AEL cases were observed after 36 months postintervention, which indicated the reason T1AEL may not have inadequate sealing due to hypovolemia. 

The concern about increasing the proximal OSR of aortic endografts originated from the effect of hemodynamic instability on aortic size. A retrospective study analyzed the difference in aortic diameters in trauma patients between admission and another stable moment, and the results suggested that the aortic diameter can decrease up to 12.6% in patients with a pulse over 130 beats/min [11]. Based on this data, current guidelines speculate that the proximal OSR may be raised to over 30% during emergent EVAR to avoid future type IA endoleak after the circulating blood volume is back to normal. Studies have suggested that CTA might underestimate aortic diameter when assessing type B aortic dissection or thoracic aorta injuries [20,21], which might contribute to late endoleak. However, recent research has indicated no significant differences in aortic measurements between intravascular ultrasound (IVUS) and CTA among non-ruptured AAA patients, particularly in proximal diameter measurements [22]. Moreover, there were no apparent differences observed in reinterventions [23]. Nevertheless, it remains unexplored whether differences exist in cases of rAAA or impending rupture AAA, and whether they have an impact on late endoleak and reinterventions. The mean MAP and heart rate of hemodynamically unstable patients in our study were 80.61 mmHg and 103.82 beats/min, which may correspond to nearly a 10% decrease in aortic diameter, according to the findings from Jonker FH and colleagues [11]. However, the change in aortic diameters may be less evident in hemodynamically unstable rAAA patients; AAA patients were much older, and the aortas were generally more calcified and stiffer than trauma patients.

We observed three T1AELs in hemodynamically unstable patients, and all of them were found after 24 months after the initial intervention. Although all three T1AELs were found in patients with a proximal OSR smaller than 30%, the late occurrence of T1AELs dispensed with the probability of inadequate sealing due to fluctuation of aortic hemodynamics, indicating that the average proximal OSR of 26% in hemodynamically unstable patients of our study was optimal. For example, for patients with an aortic neck diameter of 22 mm, choosing a 25 mm stent graft provides a standard OSR of 13.6%, which may be acceptable in elective AAA patients, but insufficient sealing of OSR smaller than 10% can occur if the collapsed aorta expands to normal size. In this circumstance, a 28 mm stent graft would be more appropriate and can provide a sufficient OSR between 15% and 27% when blood pressure fluctuates. Given the concern about the potential risk of stent migration, a proximal OSR greater than 35% may not be considered, since the diameters of some heavily atherosclerotic or calcified aortas may not be spectacular under the range of blood volume. In addition, previous experiments revealed that the dislodgement forces of thoracic aortic stents declined from 22.7 *n* to 9.0 *n* as the OSR increased from 10% to 20% [24], which suggested excessive OSR should also be avoided. Combined with the results of our study, an oversizing between 25% and 35% may be appropriate for hemodynamically unstable rAAA patients undergoing emergent EVAR. In addition, our results showed that both OSR > 20% vs. OSR ≤ 20% and OSR >30% vs. OSR ≤ 30% were not associated with the risk of reintervention in patients undergoing emergency EVAR. However, it is worth noting that the result was based on ratios rather than continuous variables. Further research is needed to determine whether a specific OSR value is associated with reintervention.

Sizing considerations should also be judged individually based on the aortic anatomy and preoperative aortic hemodynamics of the patients. A single center experience of C3 Gore Excluder stent graft suggested that a mean OSR of 23.5% was appropriate in patients with proximal aortic angulation over 60 degrees, and was not associated with an increased risk of T1AEL [25]. The latest study on EVAR for AAA using the Endurant stent graft indicates that the occurrence rate of type IA endoleaks is higher in the outside indications for use group than in those patients within the indications for use. The most common cause of endoleak in patients classified as outside indications for use is identified as having an angle or diameter issue in the proximal neck [26]. Based on their results, proper elevation of proximal OSR may be considered in patients with severe neck angulation. In addition to neck anatomy, the effect of preoperative circulating blood hemodynamics on oversizing consideration is also fundamental. The results of the IMPROVE trial suggested that the minimum threshold of 70 mmHg was too low for permissive hypotension for ruptured aneurysm, for it was associated with increased risk of 30-day mortality [27]. However, the authors did not record any increased risk of T1AEL in hemodynamically unstable patients due to inadequate sealing. Nevertheless, the results of IMPROVE trial recommended sufficient fluid resuscitation to raise mean arterial pressure before surgery, which can not only improve the survival of rAAA patients but also avoid unpredictable changes of aortic diameters due to dramatic change of blood volumes.

### Limitations

The findings of our study should be interpreted with several limitations. First of all, the vital signs of included patients were recorded on admission, and the exact value of blood pressure before stent graft implantation was not available in a retrospective setting. The potential difference of aortic hemodynamics influenced by fluid resuscitation may affect aortic diameters. Thus, future prospective studies involving the exact vital signs before stent graft implantation are needed to provide more precise insight into the effect of hemodynamic instability on OSR choice in rAAA patients. Second, as most included patients in our study were followed up by duplex ultrasound, hence it was challenging to obtain changes of aortic size before and after stent graft implantation. In spite of this, the result of T1AEL is the final observation target, which also shed light on the alterations of aortic diameters. Third, the sample size of our study is relatively small, but it is still one of the largest single-center studies regarding OSR consideration in rAAA patients. Multicenter prospective cohort studies are warranted in the future. Fourth, our center only involved the Endurant Medtronic stent graft, so the OSR recommendation from our study may not be suitable to other devices.

## 5. Conclusions

After review of the rAAA patients undergoing emergent EVAR in a single center during the past decade, this cohort study suggested a proximal OSR over 20% was associated with a decreased risk of T1AEL in hemodynamically stable patients, while there is no difference between OSR > 30% and OSR ≤ 30%. However, we cannot adequately assess the specific impact of exact OSR values on the clinical outcomes after emergency EVAR, such as whether a specific OSR value significantly affects the occurrence of type IA endoleak or the need for reintervention. Prospective studies need to further evaluate the relationship between oversizing consideration of the proximal stent and the risk of endoleak after EVAR.

## Figures and Tables

**Table 1:** Baseline characteristics of included patients.

	Hemodynamically Stable	Hemodynamically Unstable	*p*-Value
	*n* = 90	*n* = 44	
Age -y	70.41 ± 9.89	72.55 ± 8.24	0.22
MAP -mmHg	97.91 ± 12.55	80.61 ± 9.30	<0.001
HR -beats/min	77.44 ± 13.08	103.82 ± 5.96	<0.001
BMI -kg/m^2^	23.00 ± 3.15	22.93 ± 2.96	0.91
Neck diameter -mm	20.39 ± 2.41	20.43 ± 2.45	0.93
OSR -%	22% (20–27%)	25% (22–32%)	0.011
α angle -°	27.66 (12.93–45.42)	32.50 (10.77–66.73)	0.025
β angle -°	48.00 (32.00–76.00)	57.23 (31.41–78.63)	0.86
Neck length -mm	27.83 ± 12.97	25.10 ± 10.02	0.22
Neck calcification	0.01 (0.00–0.20)	0.01 (0.00–0.20)	0.76
Maximum diameter -mm	57.81 ± 14.18	63.82 ± 19.26	0.048
Within IFU	37 (41.11%)	21 (47.73%)	0.46
Gender			0.027
Male	73 (81.11%)	28 (63.64%)	
Female	17 (18.89%)	16 (36.36%)	
Hypertension	59 (65.56%)	31 (70.45%)	0.57
Diabetes	10 (11.11%)	7 (15.91%)	0.43
Pulmonary diseases	16 (17.78%)	11 (25.00%)	0.33
Peripheral artery disease	6 (6.67%)	3 (6.82%)	0.97
Stroke	6 (6.67%)	0 (0.00%)	0.97
PCI	11 (12.22%)	4 (9.09%)	0.59
CKD	8 (8.89%)	9 (20.45%)	0.059
CCI scores			0.41
Mild (≤2)	70 (77.78%)	29 (65.91%)	
Moderate (3–4)	17 (18.89%)	10 (22.73%)	
Severe (≥5)	3 (3.33%)	5 (11.36%)	
Anesthesia			0.032
Local	71 (78.89%)	27 (61.36%)	
General	19 (21.11%)	17 (38.64%)	
Severe neck angulation	24 (26.67%)	18 (40.91%)	0.10
CIAA	29 (25.66%)	9 (19.57%)	0.41

MAP = mean artery pressure, HR = heart rate, BMI = body mass index, OSR = oversizing ratio, PCI = percutaneous coronary intervention, CKD = chronic kidney disease, CCI = Charlson comorbidity index, CIAA = common iliac artery aneurysm, IFU = indications for use. Count data are expressed as *n* (%).

**Table 2:** Univariate and multivariate regression analyses of risk factors of type IA endoleak.

	Univariate Analysis		Multivariate Analysis *	
Statistics	HR (95%CI)	*p*-Value	HR (95%CI)	*p*-Value
Gender	2.76 (0.61, 12.40)	0.19		
Age	1.03 (0.94, 1.13)	0.53		
HD unstable	1.87 (0.42, 8.45)	0.41		
MAP	0.97 (0.91, 1.04)	0.39		
HR	1.01 (0.96, 1.06)	0.78		
Anesthesia	0.46 (0.05, 3.83)	0.47		
α angle	0.99 (0.97, 1.01)	0.42		
β angle	1.01 (0.99, 1.03)	0.32		
SNA	2.24 (0.43, 11.67)	0.34		
Neck diameter	1.27 (1.03, 1.56)	0.028	0.92 (0.63, 1.35)	0.67
OSR >30% vs. ≤30%	0.57 (0.06, 5.07)	0.62		
OSR >20% vs. ≤20%	0.06 (0.01, 0.31)	0.001	0.06 (0.01, 0.72)	0.026
Neck length	0.83 (0.72, 0.95)	0.009	0.74 (0.56, 0.98)	0.033
Maximum diameter	1.03 (0.98, 1.09)	0.24		
CIAA	0.63 (0.13, 3.18)	0.58		

HD = Hemodynamically, MAP = mean artery pressure, HR = heart rate, OSR = oversizing ratio, CIAA = common iliac artery aneurysm. * Adjusted for gender, age, hemodynamic instability, maximum diameter, neck angulation.

**Table 3:** Rates of adverse outcomes after emergent endovascular repair of patients with ruptured or impending rupture abdominal aortic aneurysm.

	Hemodynamically Unstable			Hemodynamically Stable		
OSR	≤30% (*n* = 30)	>30% (*n* = 14)	*p*	≤20% (*n* = 24)	>20% (*n* = 66)	*p*
Survival FU time-m	24.50 (16.00–49.25)	25.00 (9.75–53.00)	0.99	30.50 (15.00–60.50)	33.50 (11.50–69.25)	0.57
Imaging FU time-m	10.00 (1.00–39.50)	1.50 (1.25–32.50)	0.87	16.50 (5.00–29.00)	15.00 (5.25–34.50)	0.71
30-day mortality	4 (13.33%)	0 (.00%)	0.15	1 (4.17%)	1 (1.52%)	0.45
Overall survival	11 (36.67%)	6 (42.86%)	0.69	7 (29.17%)	15 (22.73%)	0.53
Reintervention	2 (6.67%)	1 (7.14%)	0.95	4 (16.67%)	6 (9.09%)	0.31
T1AEL	3 (11.11%)	0 (0.00%)	0.19	4 (16.67%)	1 (1.52%)	0.006
T1BEL	2 (7.41%)	0 (0.00%)	0.30	3 (12.50%)	4 (6.06%)	0.31
T2EL	5 (18.52%)	2 (14.29%)	0.73	6 (25.00%)	14 (21.21%)	0.70

OSR = oversizing ratio, FU = follow-up, T1AEL = type IA endoleak, T1BEL = type IB endoleak, T2EL = type II endoleak.

## Data Availability

The dataset generated and analyzed is available from the corresponding author upon reasonable request.

## Supplementary Materials

The following supporting information can be downloaded at: https://www.mdpi.com/article/10.3390/jcm12237500/s1, Table S1: Strengthening the reporting of observational studies in epidemiology (STROBE) checklist of cohort studies; Table S2: Univariate and multivariate analyses of outcomes of interest in patients undergoing emergent endovascular aortic repair.
